# Modified Sullivan-type segment III hepatojejunostomy for radiologically presumed unresectable hilar cholangiocarcinoma: a technical case report

**DOI:** 10.1093/jscr/rjag906

**Published:** 2026-10-12

**Authors:** Mirko Lombardi, Bahati G Kamwezi, Tulia M Hadi, Lusekelo W Mwalukambwe, Jonathan Mathayo, Shama V Mkini, Tresphory Boniphace Kamushaga, Agnes L Mlawa

**Affiliations:** Department of Surgery, Consolata Hospital Ikonda, Makete District, Njombe Region, PO Box 700, Njombe, Tanzania; Department of Surgery, Consolata Hospital Ikonda, Makete District, Njombe Region, PO Box 700, Njombe, Tanzania; Department of Surgery, Consolata Hospital Ikonda, Makete District, Njombe Region, PO Box 700, Njombe, Tanzania; Department of Surgery, Consolata Hospital Ikonda, Makete District, Njombe Region, PO Box 700, Njombe, Tanzania; Department of Radiology, Consolata Hospital Ikonda, Makete District, Njombe Region, PO Box 700, Njombe, Tanzania; Department of Surgery, Consolata Hospital Ikonda, Makete District, Njombe Region, PO Box 700, Njombe, Tanzania; Department of Surgery, Consolata Hospital Ikonda, Makete District, Njombe Region, PO Box 700, Njombe, Tanzania; Department of Surgery, Consolata Hospital Ikonda, Makete District, Njombe Region, PO Box 700, Njombe, Tanzania

**Keywords:** hilar cholangiocarcinoma, segment III hepatojejunostomy, Sullivan modification, palliative biliary bypass, low-resource setting

## Abstract

Hilar cholangiocarcinoma may present with advanced biliary obstruction requiring palliation. An 80-year-old frail woman with jaundice and severe pruritus had contrast-enhanced computed tomography findings radiologically consistent with Bismuth–Corlette type IV hilar cholangiocarcinoma. No tissue diagnosis was obtained. Cancer antigen 19-9, carcinoembryonic antigen, alkaline phosphatase, magnetic resonance cholangiopancreatography, endoscopic retrograde cholangiopancreatography, and percutaneous transhepatic cholangiography were unavailable at the treating hospital and were not performed. Because referral and non-operative biliary drainage were not feasible and the patient requested active symptom-directed treatment, a modified Sullivan-type segment III hepatojejunostomy was performed. Operative time was 85 min and blood loss 100 ml. Pruritus resolved and bilirubin decreased from 191.78 to 127.81 μmol/l by postoperative Day 10. The patient died ~2 months later from disease progression. The procedure therefore provided short-term symptomatic improvement and partial biochemical improvement only, illustrating a potential palliative option when standard drainage modalities are inaccessible.

## Introduction

Hilar cholangiocarcinoma commonly presents with obstructive jaundice and may be unsuitable for curative resection at diagnosis [1–3]. Endoscopic or percutaneous biliary drainage is generally preferred for palliation where available [4]. In resource-limited settings, however, these options and referral may be inaccessible. Segment III hepatojejunostomy, derived from the Longmire procedure and its modifications, can provide intrahepatic biliary drainage without formal hilar dissection [5–10]. We report its use in a frail patient with radiologically presumed advanced hilar cholangiocarcinoma in rural Tanzania.

## Case report

An 80-year-old woman presented with 3 weeks of progressive jaundice, severe pruritus, anorexia, ~8 kg weight loss, dark urine, pale stools, and right upper-quadrant pain. She was deeply jaundiced, moderately malnourished, and mildly dehydrated. Total bilirubin was 191.78 μmol/l (reference 2.00–21.00), direct bilirubin 151.64 μmol/l (0–5.13), alanine aminotransferase 50.5 U/l (0–45), aspartate aminotransferase 108.3 U/l (0–31), hemoglobin 10.2 g/dl, and creatinine 50.9 μmol/l (44–88). Alkaline phosphatase was not measured because the assay was unavailable locally.

Contrast-enhanced computed tomography (CT) showed a hilar lesion with marked bilateral intrahepatic biliary dilatation and involvement of both right and left ductal systems extending into secondary branches, radiologically corresponding to a Bismuth–Corlette type IV pattern (Fig. 1). Cancer antigen 19-9 and carcinoembryonic antigen assays, magnetic resonance cholangiopancreatography, endoscopic retrograde cholangiopancreatography, and percutaneous transhepatic cholangiography were unavailable at the treating hospital and were not performed. No biopsy or cytology was obtained because histologic confirmation was not expected to alter the immediate palliative plan in this frail patient. There was no formal multidisciplinary tumor board; the case was reviewed locally by the surgeon, radiologist, and physician. At operation, a hard advanced hilar mass was palpable. The disease extent and frailty made curative resection inappropriate in this clinical setting.

**Figure 1:**
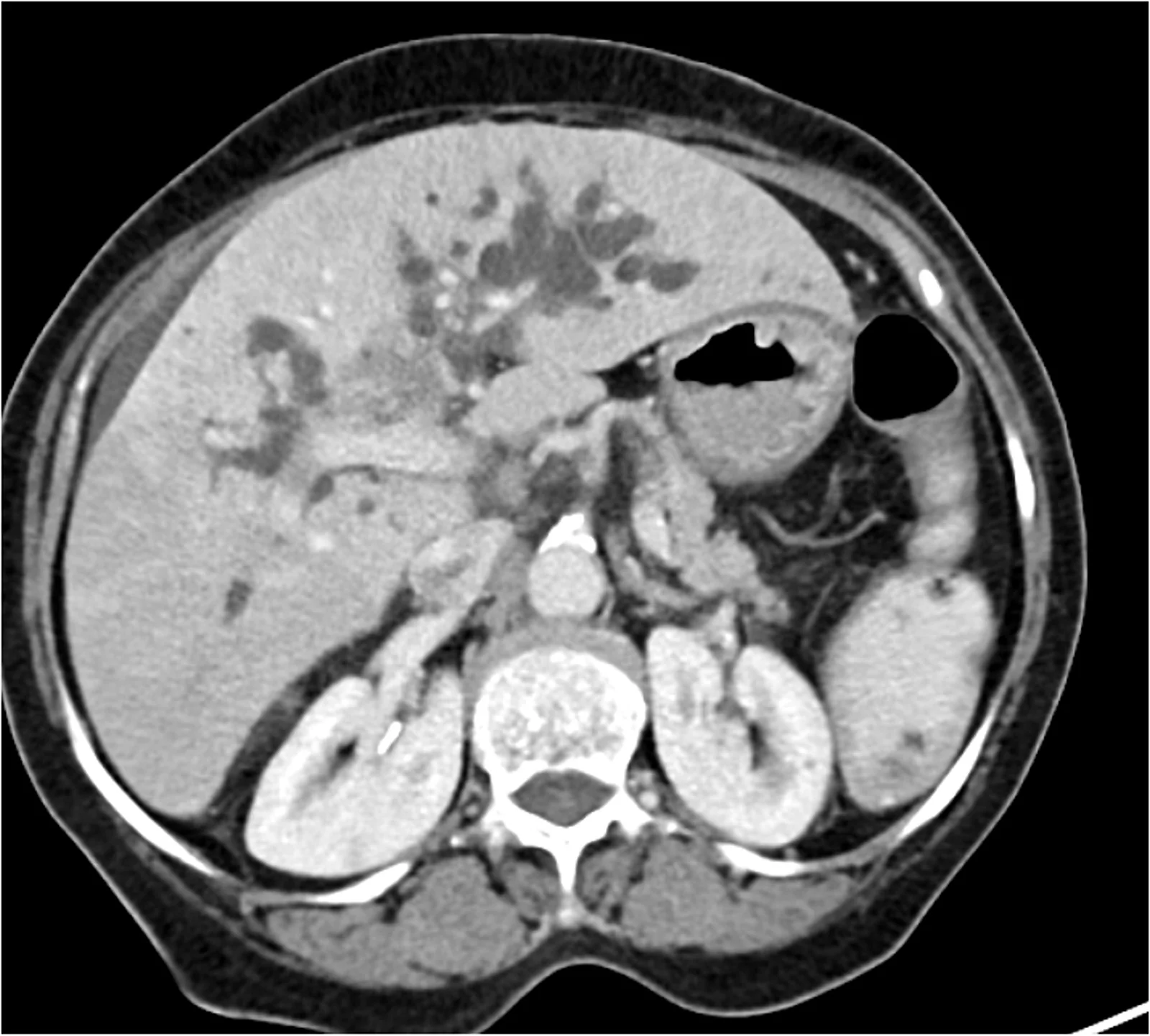
Contrast-enhanced axial CT demonstrating a hilar lesion with marked bilateral intrahepatic biliary ductal dilatation and involvement of both right and left ductal systems extending into secondary branches, radiologically corresponding to a Bismuth–Corlette type IV pattern.

Interventional radiology and therapeutic biliary endoscopy were unavailable locally, and referral was not feasible because of financial and logistical barriers and the patient’s physical condition. Her principal goal was relief of severe pruritus and jaundice-related symptoms, and she firmly requested active treatment. Expected benefit, perioperative risk, and the possibility of limited or absent durable benefit were discussed. A limited surgical bypass was therefore offered after informed consent.

Through a small right subcostal incision curved ~3 cm across the midline, a 3 × 6 cm wedge of segment III was resected to expose peripheral bile ducts. Two ducts were identified; one ~2 mm duct was cannulated with a 5-Fr, 12-cm feeding catheter. A Roux limb longer than 50 cm was fashioned from the second jejunal loop. The jejunojejunostomy was hand-sewn end-to-side in two layers with 2–0 absorbable polyglactin 910, and the limb was passed transmesocolically.

The hepatojejunostomy was constructed between the exposed bile-draining hepatic surface and the Roux limb using continuous 4–0 polypropylene, reinforced with interrupted U-stitches tied on the intestinal side to reduce parenchymal tearing. The catheter was incorporated into the anastomosis, anchored at its inferior margin with 4–0 absorbable suture, left entirely internal, and expected to pass spontaneously without planned retrieval. A drain was left adjacent to the anastomosis. Operative time was 85 min and estimated blood loss 100 ml (Figs 2 and 3).

**Figure 2:**
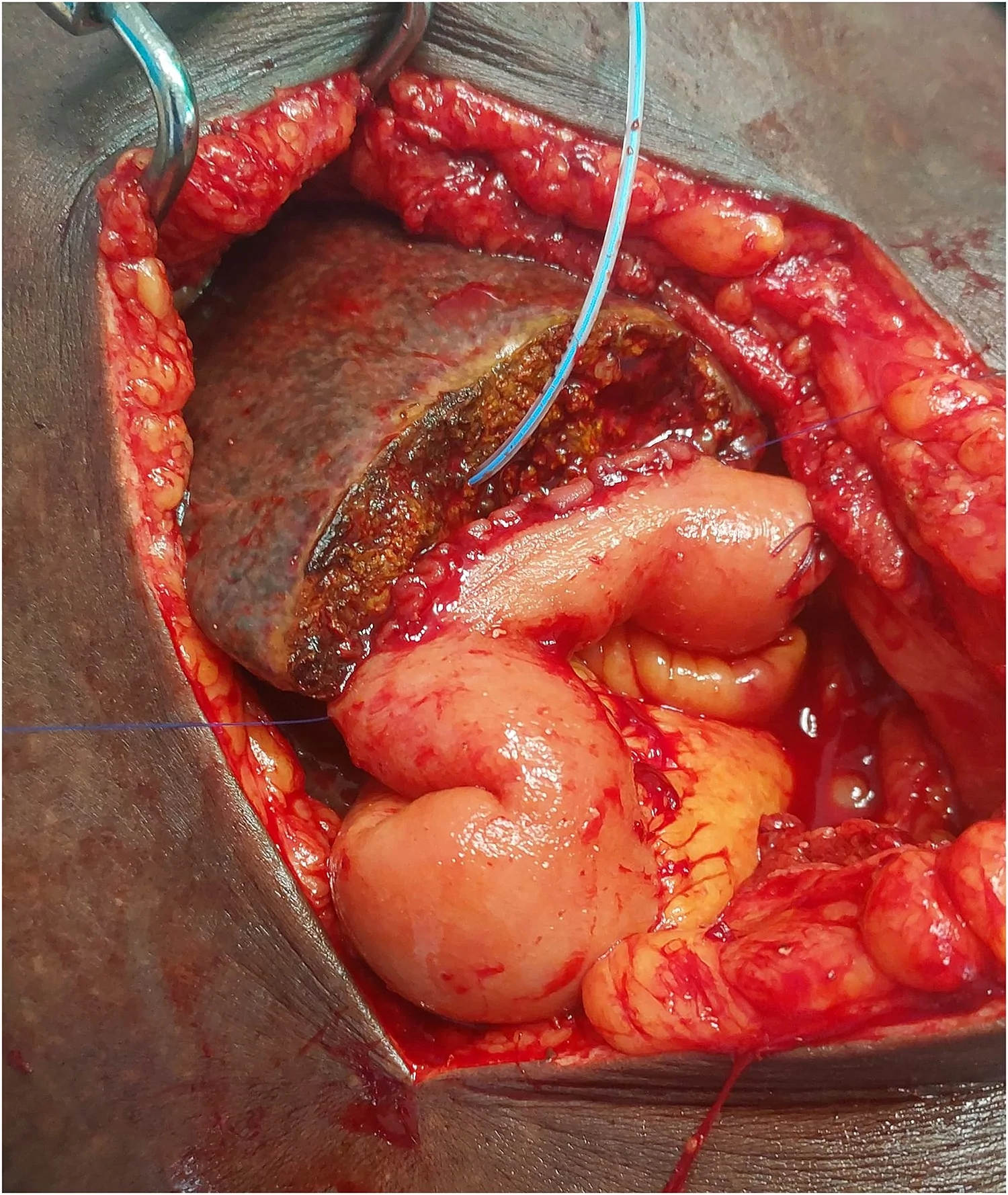
Operative view after limited segment III wedge resection showing the exposed hepatic transection surface and peripheral bile ducts; a 5-Fr feeding catheter is inserted into a peripheral bile duct before construction of the hepatojejunostomy.

**Figure 3:**
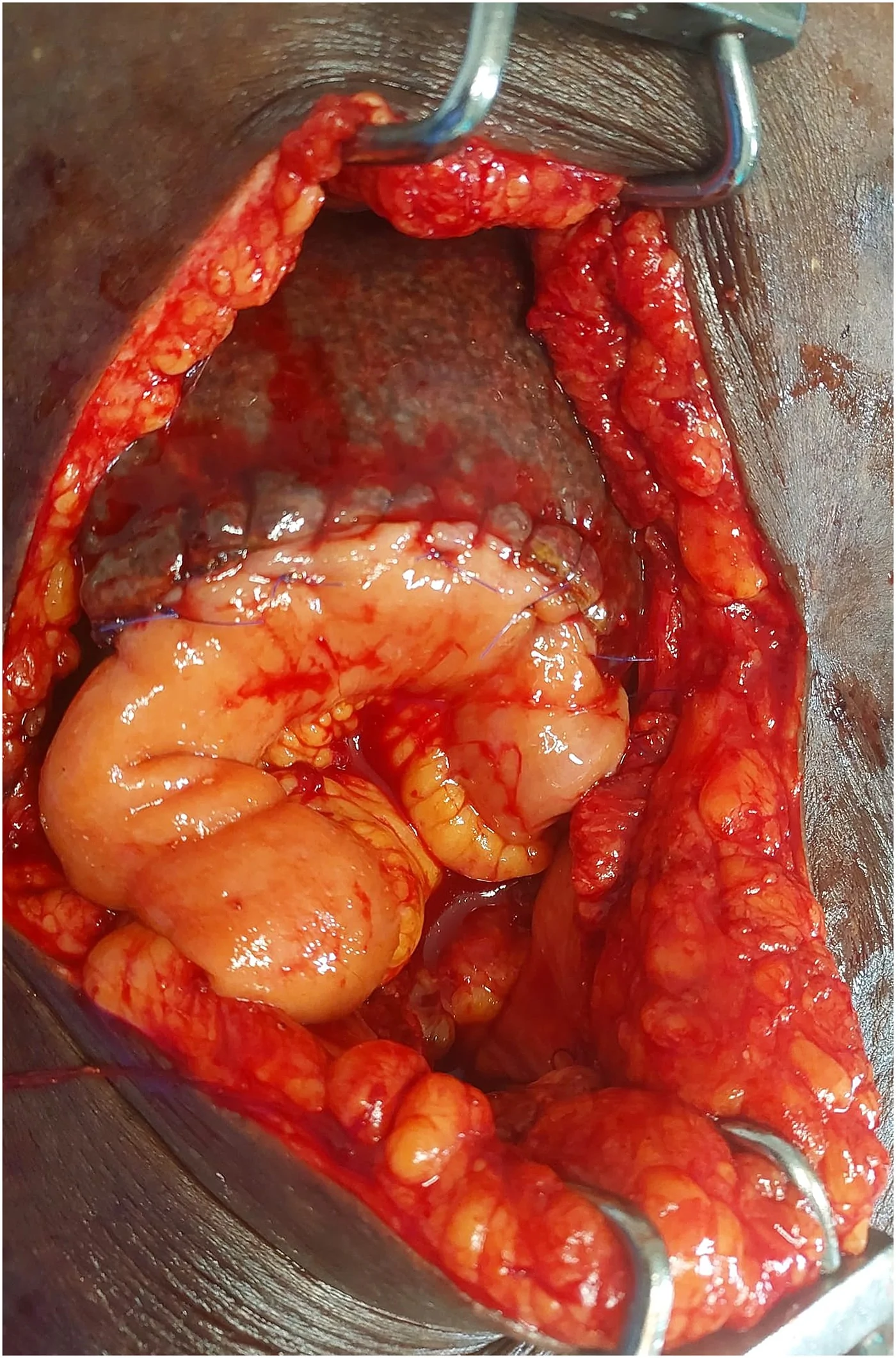
Completed modified Sullivan-type segment III hepatojejunostomy; the Roux-en-Y jejunal limb is sutured directly to the exposed segment III hepatic surface, creating a broad biliary drainage interface without a formal duct-to-mucosa anastomosis.

Ceftriaxone 2 g once daily was administered for 3 days, beginning on the day of surgery; the first dose was given 2 h before skin incision. The drain was monitored for bile leakage and removed on postoperative Day 5 when no leak was observed. Bowel function returned by Day 3 and a normal diet was resumed by Day 5. Pruritus rapidly decreased and disappeared, appetite improved, and total bilirubin fell to 127.81 μmol/l by postoperative Day 10. The patient was discharged in stable condition and was able to perform light domestic activities. She died ~2 months after surgery at another healthcare facility from progression of the underlying malignancy; later biochemical testing was unavailable.

## Discussion

The procedure was associated with short-term symptomatic improvement and partial biochemical improvement, not documented durable biliary decompression. The limited operation avoided complex hilar dissection and may be considered only in selected circumstances where standard endoscopic or percutaneous palliation and referral are not feasible [4, 11].

The internal 5-Fr catheter was intended to support early drainage and then pass spontaneously. Migration is possible, and obstruction cannot be completely excluded, although it was considered unlikely because of the small caliber and 12-cm length. This catheter strategy therefore requires caution and cannot be recommended from a single case.

This report is limited by the absence of tissue confirmation, tumor markers and additional cholangiographic imaging, short biochemical follow-up, unknown long-term anastomotic patency and catheter fate, and death ~2 months after surgery. It supports technical feasibility and short-term symptom relief only; no conclusions can be drawn regarding safety, reproducibility, survival benefit, or long-term efficacy. The Sullivan-type segment III approach may nevertheless remain a pragmatic palliative option in carefully selected patients when less invasive biliary drainage is genuinely inaccessible [12–14].
